# Association of macular thickness with parapapillary atrophy in myopic eyes

**DOI:** 10.1186/s12886-020-01362-8

**Published:** 2020-03-06

**Authors:** Helong Piao, Yue Guo, Jun Young Ha, Mi Sun Sung, Sang Woo Park

**Affiliations:** 1 Department of Ophthalmology and Research Institute of Medical Sciences, Chonnam National University Medical School and Hospital, 42 Jebong-ro, Dong-gu, Gwangju, 61469 South Korea; 2 Department of Ophthalmology, Yanbian University Hospital, Yanji, Jilin China; 3 Eye Hospital and School of Ophthalmology and Optometry, Wenzhou Medical University, Wenzhou, Zhejiang China

**Keywords:** Macular thickness, Axial length, Myopia, β-Parapapillary atrophy with Bruch’s membrane, β-Parapapillary atrophy without Bruch’s membrane

## Abstract

**Background:**

To investigate whether macular structure could be affected by axial elongation and to determine the association between macular intraretinal thickness and the microstructure of β-zone parapapillary atrophy (PPA) in myopic eyes.

**Methods:**

The study recruited 113 healthy myopic subjects (113 eyes). Images of the macula, subfoveal choroid, and optic nerve head were acquired using spectral-domain optical coherence tomography (SD-OCT). An automatic segmentation algorithm was used to segment the macular images into 7 intraretinal layers. PPA widths with and without Bruch’s membrane (PPA_+BM_ and PPA_-BM_, respectively) were evaluated. Linear regression analysis was performed to evaluate the association between macular intraretinal thickness and axial length and the microstructure of PPA.

**Results:**

An increase in axial length was associated with a decrease in whole macular thickness of the peripheral region and an increase in whole macular thickness of the central region. Thickness alterations of the macular intraretinal layers were most apparent in the peripheral region. A significant correlation was found between PPA_-BM_ width and macular intraretinal layer thickness, whereas no significant correlation was found between PPA_+BM_ width and macular intraretinal layer thickness. Moreover, both PPA_+BM_ and PPA_-BM_ widths significantly correlated with subfoveal choroidal thickness.

**Conclusions:**

Macular intraretinal layer thickness may be affected by PPA_-BM_ width. These findings indicate that the microstructure of PPA should be considered when evaluating the macula in patient with myopia and glaucoma.

## Background

Parapapillary atrophy (PPA) is common in myopic and glaucomatous eyes, which is classified into the peripheral α-zone and central β-zone on fundus photography. The α-zone is an irregular pigmentation of the retinal pigment epithelium (RPE), while the β-zone is considered atrophy of the choroid and RPE. On the basis of the location of Bruch’s membrane (BM) termination, β-zone PPA can be classified into β-parapapillary atrophy with and without BM (PPA_+BM_ and PPA_-BM_). The region with BM but without the RPE is PPA_+BM_, and the region with neither the RPE nor BM is PPA_-BM_ (or in other words γ-zone PPA) [1]. Several clinic-based studies have shown that PPA_+BM_ was mostly associated with glaucoma, older age, and myopia. However, PPA_-BM_ was associated with a longer axial length but not with glaucoma [2–5]. A previous study showed that optic nerve head (ONH) tilt was associated with PPA_+BM_ and PPA_-BM_ widths, which suggested that the microstructure of PPA might reflect the myopic remodeling of the ONH [6]. However, PPA’s formation and significance are not fully understood.

Myopia is a very common ocular disorder worldwide. In addition to the elongation of axial length, myopic eyes exhibit a series of pathophysiological changes, including thinning of the retina, choroid, and sclera [7–13]. With the progression of myopia, the potential risks of visual impairment become significantly higher and include retinal detachment, posterior staphyloma, cataract, choroidal neovascularization, and macular hole [14, 15]. Several cross-sectional studies have suggested that myopic eyes were more susceptible to glaucoma than normal eyes [16, 17]. Therefore, understanding the mechanism of myopia and its underlying effect on the retina will be useful for the earlier diagnosis of myopia-related diseases.

Spectral-domain optical coherence tomography (SD-OCT) provides high-resolution images and enables the visualization of detailed morphological changes in each macular intraretinal layer and ONH [18]. In macula, evaluation of each intraretinal layer can give us the valuable information for the diagnosis and monitoring of several eye diseases including macular edema, glaucoma, and optic neuropathy and also the prediction of its variability is helpful for managing retinal diseases [19–21]. Of note, previous studies have reported that macular evaluation can be a valuable clinical parameter in managing patients with early to advanced glaucoma [22–25]. Jung et al. [22] reported that the evaluation of macular ganglion cell-inner plexiform layer (mGCIPL) thickness showed the similar diagnostic power to that of parapapillary retinal nerve fiber layer (pRNFL) thicknesses in patient with normal tension glaucoma or primary open angle glaucoma. Sung et al. [25] showed that the assessment of macular thickness was useful parameter for detection of progression in patients with advanced glaucoma.

Previous studies have investigated the association between the age or several ocular parameters and thickness of macular intraretinal layers [9, 26–28]. However, these studies yielded conflicting results. In this study, we hypothesized that the changes in the ONH might be associated with the alterations of the posterior pole. Since the development of β-zone PPA (especially PPA_-BM_) in myopic eyes is known to be associated with the mechanical stretching of eyeball during axial elongation, we thought that there might be some associations between the β-zone PPA and macular thickness in myopic eyes. Given the clinical significance of β-zone PPA and the importance of macular evaluation in myopic glaucoma, investigating the relationships between PPA and macular layer might be important. The purpose of this study was to determine whether macular intraretinal thickness was affected by axial elongation and to investigate the association between macular intraretinal thickness and the microstructure of PPA in myopic eyes.

## Methods

### Subjects

The study subjects were recruited from the Young Myopia Study of Chonnam National University Hospital, which is an ongoing cross-sectional study that commenced in January 2018. The present study was conducted according to the tenets of the Declaration of Helsinki and was approved by the Institutional Review Board of Chonnam National University Hospital. All patients provided written informed consent prior to enrollment in the study.

The Young Myopia Study enrolled consecutive participants who visited the general eye clinic for medical check-ups and met all the inclusion criteria and none of the exclusion criteria. Ocular examinations included measurements of best-corrected visual acuity (BCVA), intraocular pressure (IOP) using Goldmann applanation tonometry, autorefractometry, slit-lamp examination, and anterior chamber angle examination using gonioscopy. Central corneal thickness, keratometry, and axial length were measured using optical low-coherence reflectometry (Lenstar; Haag-Streit AG, Koeniz, Switzerland); ONH and pRNFL examinations were performed using color stereoscopic disc photography and red-free RNFL fundus photography; and visual field testing was performed using the Swedish Interactive Threshold Algorithm standard 30–2 perimetry with a Humphrey Field Analyzer (Carl Zeiss Meditec Inc., Dublin, CA, USA).

The inclusion criteria used in this study were as follows: ophthalmologically healthy subjects aged between 20 and 35 years old, spherical equivalent (SE) between − 12.0 and − 0.5 diopters, astigmatism within ±2 diopters, BCVA of 20/25 or better, IOP ≤ 21 mmHg, normal ONHs on stereoscopic photographs (without a narrowed neuroretinal rim, abnormal tilt of the ONH, parapapillary hemorrhage, or paleness), and the absence of any pRNFL abnormalities on red-free fundus photographs. Patients with visual field defects, a history of intraocular surgery, glaucoma, neurologic diseases, refractive surgery, other systemic diseases that affected ocular media opacities, or low-quality OCT images were excluded.

### Spectral-domain optical coherence tomography

All subjects underwent traditional OCT imaging using SD-OCT (Heidelberg Spectralis SD-OCT; Spectralis software version 6.9.4; Heidelberg Engineering GmbH, Heidelberg, Germany). One skilled operator performed all OCT scans. To avoid the diurnal variation of retinal thickness with SD-OCT, all subjects had retinal thickness measured at 10–12 AM. Ocular magnification effect was corrected using the formula provided by the manufacturer on the basis of the results of corneal radius, axial length, and focus setting during image acquisition. OCT images with insufficient quality (typically truncated B-scans and quality score < 30) were excluded.

**Fig. 1:**
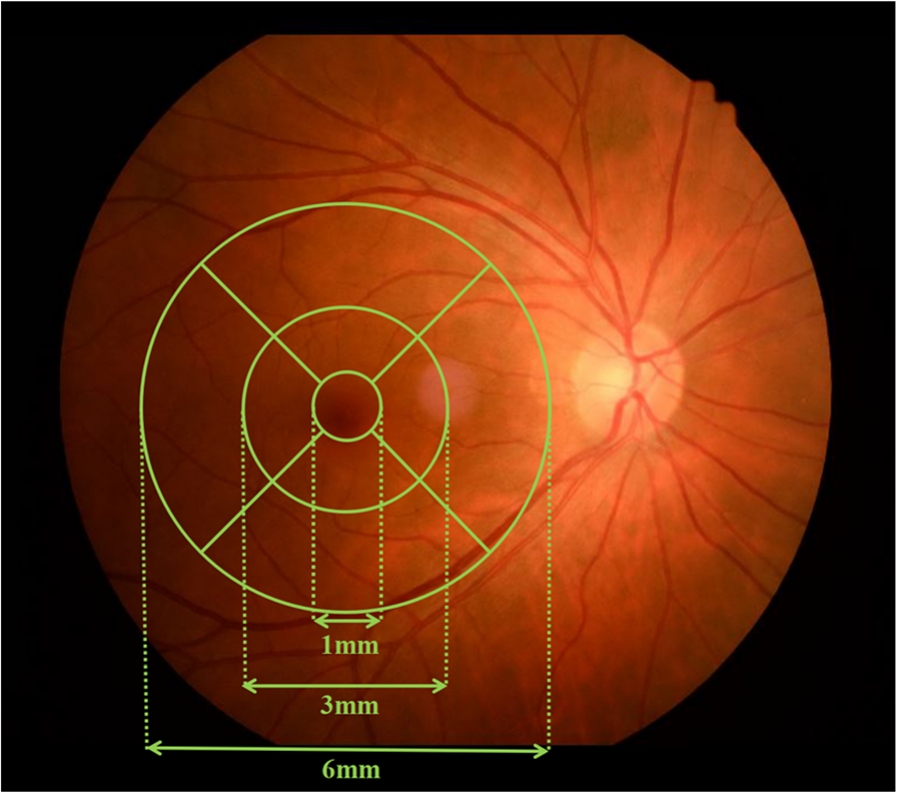
Analysis of the macular region by using Heidelberg Spectralis spectral-domain optical coherence tomography. The macula is subdivided into 3 concentric circles. The central region is a circle with a 1-mm diameter centered on the fovea. The pericentral region around the central region has a 3-mm diameter centered on the fovea. The peripheral region around the pericentral and central regions has a 6-mm diameter centered on the fovea

**Fig. 2:**
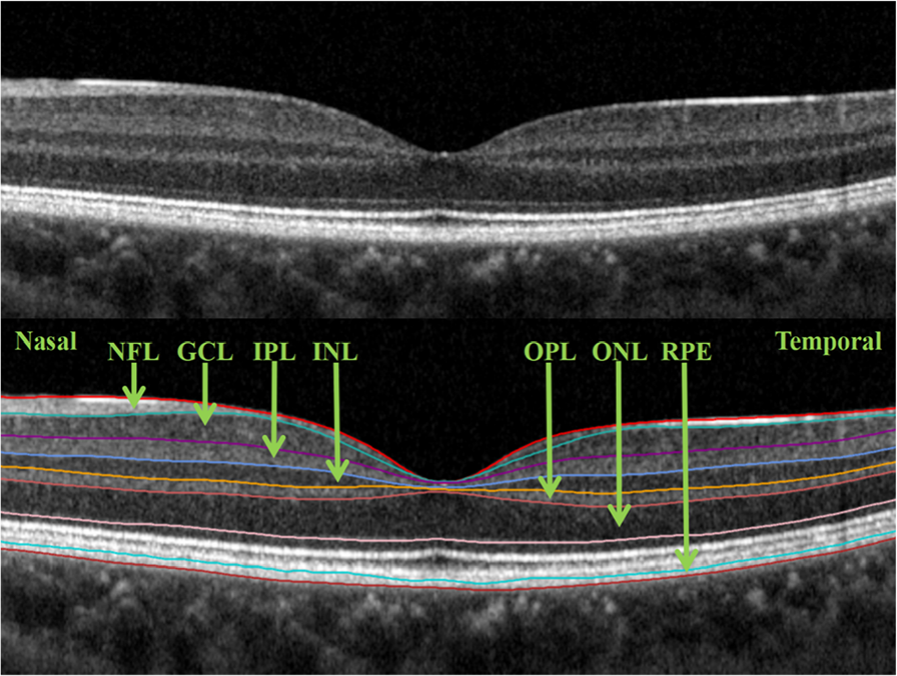
A cross-sectional image acquired using Heidelberg Spectralis spectral-domain optical coherence tomography (upper panel). The automatic segmentation algorithm divides the macula into 7 layers (lower panel). The 7 intraretinal layers include the RNFL = retinal nerve fiber layer; GCL = ganglion cell layer; IPL = inner plexiform layer; INL = inner nuclear layer; OPL = outer plexiform layer; ONL = outer nuclear layer; and RPE = retinal pigment epithelium

The macular cube volumetric scans with 49 raster lines, 20 × 20°, interscan distance of 120 μm between the 49 B-scans and centered on the fovea were acquired after mydriasis. The built-in software for automated segmentation of the intraretinal layers was applied, followed by manual inspection for obvious segmentation errors. The automatic segmentation algorithm divided the macular region into 9 sectors (Fig. 1) and 7 layers (Fig. 2), and they were named according to the guidelines of the Early Treatment Diabetic Retinopathy Study [29] and the International Nomenclature for Optical Coherence Tomography Panel [30]. For analysis, the macular region was divided into 3 concentric circles. The central region was a circle with a 1-mm diameter centered on the fovea. The pericentral region around the central region had a 3-mm diameter centered on the fovea. The peripheral region around the pericentral and central regions had a 6-mm diameter centered on the fovea. The 7 intraretinal layers included the RNFL, ganglion cell layer (GCL), inner plexiform layer (IPL), inner nuclear layer (INL), outer plexiform layer (OPL), outer nuclear layer (ONL), and RPE. The algorithm set the distance between the internal limiting membrane (ILM) and the middle of the RPE as the full retinal layer thickness.

### Optic nerve head measurements

The Heidelberg Spectralis OCT enhanced depth imaging (EDI) mode was used for the other ONH measurements. The ONH was scanned by centering a 15° × 10° rectangular scan on the ONH. Each OCT volume consisted of 49 serial horizontal B-scans (4.5-mm-long lines; 50 images averaged) spaced at approximately 63-μm intervals. Infrared (IR) fundus images were acquired simultaneously by using a confocal scanning laser ophthalmoscope. Three sections that passed through the ONH in the horizontal scans and upper and lower scans were selected, and all the study parameters were measured in each of these frames by 2 independent examiners (M.S.S and H.P), in a masked fashion.

Temporal β-zone PPA margin, Bruch’s membrane opening (BMO), and disc margin were determined using IR fundus images. Temporal β-zone PPA margin and γ-zone PPA margin were also evaluated using IR fundus images. According to the location of BM termination, PPA was further divided into PPA_+BM_ and PPA_-BM_; PPA_+BM_ width was defined as the distance from the beginning of the RPE to BMO, and PPA_-BM_ width was defined as the distance from the temporal disc margin to the beginning of BM. The measurement was performed using a previously described method [31, 32].

### Subfoveal choroidal thickness measurements

Subfoveal choroidal thickness was measured using the EDI technique with a 12-mm-length scan running through the fovea. The vertical distance from the hyperscattering outer border of the RPE to the inner border of the sclera at the fovea was defined as subfoveal choroidal thickness. Images with a signal strength below 70 or cases wherein the RPE and chorioscleral interface were not clearly identified were excluded from the analysis. The average of data from 2 independent examiners (M.S.S and H.P) were used in this study. In cases of disagreement, an open adjudication with a senior grader (S.W.P) was performed.

### Statistical analysis

IBM SPSS Statistics for Windows, Version 25.0 (IBM Corp., Armonk, NY, USA) was used for statistical analysis. Multiple linear regression analysis was applied to determine the associations of macular thickness of each intraretinal layer with axial length and PPA_+BM_ and PPA_-BM_ widths. Coefficients with 95% confidence intervals were presented. *P* values were adjusted to control the false discovery rate using the Benjamini-Hochberg procedure [33]. *P* values < 0.05 were considered statistically significant.

## Results

The demographic and ocular characteristics of 113 eyes of 113 patients (74 men and 39 women) are shown in Table 1. The mean age of the patients was 26.8 ± 3.2 years (range, 20–35 years). The mean axial length and SE were 26.0 ± 1.5 mm (range, 22.8–29.8 mm) and − 5.0 ± 2.9 diopter (range, − 11.25 to − 0.5 diopter), respectively. The mean IOP was 13.6 ± 1.9 mmHg (range, 9–19 mmHg). Of the 113 subjects, 33 are low myopia, 41 are moderate myopia, 39 are high myopia, accounting for 29, 36 and 35% of the total, respectively.

**Table 1:** Demographics and ocular parameters of study subjects

	Number of subjuects (***n*** = 113)		
	Mean	SD	Range
Age, y	26.8	3.2	20–35
Sex, male/female	74/39		
Axial length, mm	26.0	1.5	22.8–29.8
IOP, mm Hg	13.6	1.9	9–19
Spherical equivalent, diopter	−5.0	2.9	−11.25 – − 0.5
Average keratometry, diopter	42.8	1.1	39.9–45.7
Central corneal thickness, μm	554.3	36.2	484–674
PPA_+BM_ width, μm	180.5	174.8	0–868
PPA-_BM_ width, μm	284.5	216.3	0–706
Subfoveal choroidal thickness, μm	268.0	85.4	80–416
Average pRNFL thickness, μm	89.7	8.3	72–109

*IOP* Intraocular pressure, *PPA*_*+BM*_ β-parapapillary atrophy with Bruch’s membrane, *PPA-*_*BM*_ β-parapapillary atrophy without Bruch’s membrane, *pRNFL* Parapapillary retinal nerve fiber layer

Table 2 shows the results of the multiple linear regression analysis of the correlation between whole macular thickness and each intraretinal layer thickness and axial length. In evaluation of whole macular thickness, the central region (*P* = 0.006, unstandardized regression coefficient B: 3.585) increased with axial elongation. However, the peripheral region (*P* < 0.001, B: − 3.313) decreased with axial elongation.

**Table 2:** Associations of the thickness of whole macula and each macular layer with axial length in myopic eyes

	Axial Length		
	B (95% CI)	***P*** Value^a^	***P*** Value^**b**^
Central region			
OPL	1.474 (0.726–2.222)	**< 0.001**	**< 0.001**
ONL	− 0.983 (− 2.381–0.415)	0.166	0.229
Whole	3.585 (1.215–5.955)	**0.003**	**0.006**
Pericentral region			
NFL	0.540 (0.339–0.740)	**< 0.001**	**< 0.001**
GCL	0.056 (− 0.342–0.455)	0.779	0.825
IPL	− 0.023 (− 0.258–0.212)	0.846	0.846
INL	− 0.156 (− 0.508–0.196)	0.382	0.429
OPL	0.341 (− 0.271–0.952)	0.272	0.343
ONL	−1.127 (− 2.240 – − 0.014)	**0.047**	0.070
Whole	− 0.952 (− 2.712–0.807)	0.286	0.343
Peripheral region			
NFL	0.533 (0.118–0.948)	**0.012**	**0.023**
GCL	−1.051 (− 1.362 – − 0.740)	**< 0.001**	**< 0.001**
IPL	− 0.771 (− 1.013 – − 0.528)	**< 0.001**	**< 0.001**
INL	− 0.489 (− 0.727 – − 0.250)	**< 0.001**	**< 0.001**
OPL	−0.263 (− 0.487 – − 0.038)	**0.022**	**0.036**
ONL	− 0.903 (− 1.615 – − 0.192)	**0.013**	**0.023**
Whole	−3.313 (− 4.849 – − 1.777)	**< 0.001**	**< 0.001**
Subfoveal choroid	−29.116 (− 38.207 – − 20.024)	**< 0.001**	**< 0.001**

*B* Unstandardized coefficient B, *NFL* Nerve fiber layer; *GCL* Ganglion cell layer, *IPL* Inner plexiform layer, *INL* Inner nuclear layer, *OPL* Outer plexiform layer, *ONL* Outer nuclear layer

^a^*P* values adjusted for age and sex

^b^*P* values adjusted for age and sex. They were adjusted with Benjamini-Hochberg procedure

Values with statistical significance are shown in bold

In the central region, OPL was positively correlated with axial length (*P* < 0.001, B: 1.474). In the peripheral region, all intraretinal layer thicknesses were negatively correlated with axial length (*P* < 0.001, B: -1.051; *P* < 0.001, B: -0.771; *P* < 0.001, B: -0.489; *P* = 0.036, B: -0.263; *P* = 0.023, B: − 0.903; and *P* < 0.001, B: − 3.313 for GCL, IPL, INL, OPL, ONL, and whole macular thicknesses, respectively), except for RNFL thickness (*P* = 0.023, B: 0.533). In addition, subfoveal choroidal thickness (*P* < 0.001, B: − 29.116) significantly decreased with axial elongation. Among the 3 macular regions, the peripheral region was the most affected by axial elongation.

**Fig. 3:**
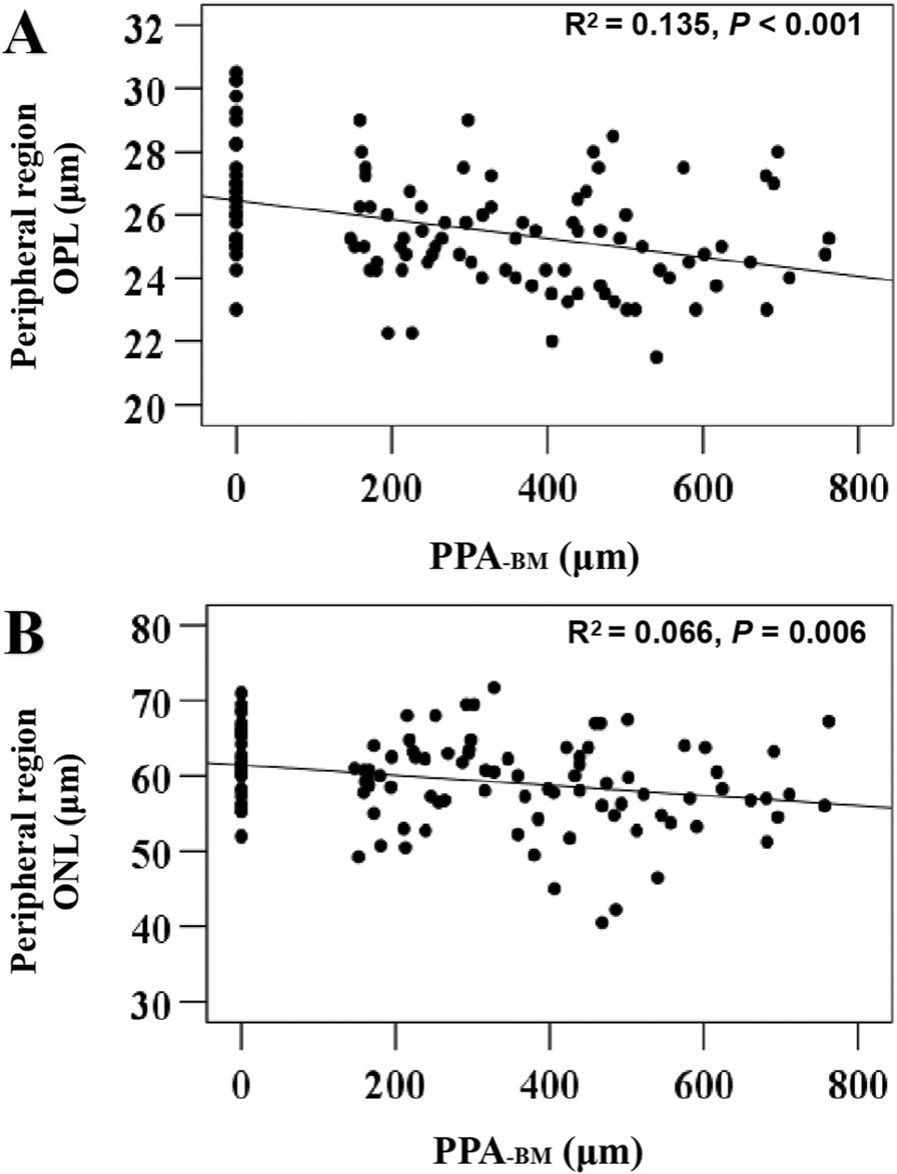
Scatter plots showing the relationship between macular intraretinal layer thickness and β-parapapillary atrophy with Bruch’s membrane (PPA_-BM_) width. **a** Association between outer plexiform layer (OPL) thickness in the peripheral region and PPA_-BM_ width. **b** Association between outer nuclear layer (ONL) thickness in the peripheral region and PPA_-BM_ width

Table 3 shows the relationships between whole macular thickness and each intraretinal layer thickness and the microstructure of PPA, which were analyzed using multiple linear regression analysis. No significant correlation was found between PPA_+BM_ width and whole macular thickness and each intraretinal layer thickness. In contrast, PPA-_BM_ width had correlations with macular thicknesses. In the peripheral region, OPL, ONL, and whole macular thicknesses were negatively correlated with PPA_-BM_ width (*P* = 0.005, B: -0.003; *P* = 0.033, B: − 0.007; and *P* = 0.047, B: − 0.020 for OPL, ONL, and whole macular thicknesses, respectively). In the pericentral region, INL and whole macular thicknesses were negatively correlated with PPA-_BM_ width (*P* = 0.047, B: − 0.003; and *P* = 0.032, B: − 0.017 for INL and whole macular thicknesses, respectively). Subfoveal choroidal thickness was significantly correlated with both PPA_+BM_ (*P* = 0.007, B: − 0.183) and PPA-_BM_ widths (*P* < 0.001, B: − 0.194). Figure 3 shows the association between intraretinal layer thickness (of the OPL and ONL in the peripheral region) and PPA_-BM_ width.

**Table 3:** Associations of the thickness of whole macula and each macular layer with microstructure of PPA

	PPA_**+BM**_				PPA-_**BM**_			
	B (95% CI)	***P*** value^a^	***P*** value^b^	***P*** value^c^	B (95% CI)	***P*** value^a^	***P*** value^b^	***P*** value^c^
Central region								
OPL	0.004 (−0.003–0.011)	0.210	0.980	0.980	0.010 (− 0.005–0.016)	< **0.001**	**0.018**	**0.002**
ONL	− 0.005 (− 0.018–0.007)	0.387	0.680	0.803	−0.016 (− 0.026 – − 0.007)	**0.001**	**0.003**	**0.006**
Whole	0.006 (−0.016–0.027)	0.599	0.600	0.831	0.004 (− 0.014–0.021)	0.685	0.266	0.363
Pericentral region								
NFL	0.001 (−0.001–0.003)	0.378	0.526	0.789	0.001 (−0.001–0.002)	0.308	0.187	0.306
GCL	−0.002 (− 0.006–0.001)	0.211	0.154	0.468	−0.002 (− 0.005–0.001)	0.161	0.081	0.162
IPL	− 0.002 (− 0.004–0.000)	0.097	0.100	0.468	−0.001 (− 0.002–0.001)	0.269	0.277	0.363
INL	− 0.003 (− 0.006–0.000)	0.094	0.140	0.468	−0.003 (− 0.005–0.000)	**0.023**	**0.040**	**0.047**
OPL	0.000 (− 0.005–0.006)	0.931	0.824	0.927	0.000 (− 0.004–0.004)	0.886	0.303	0.412
ONL	0.001 (− 0.008–0.011)	0.772	0.342	0.559	−0.009 (− 0.016 – − 0.001)	0.025	0.129	0.232
Whole	−0.003 (− 0.018–0.012)	0.673	0.935	0.980	−0.017 (− 0.028 – − 0.005)	**0.005**	**0.011**	**0.032**
Peripheral region								
NFL	0.004 (0.001–0.008)	0.056	0.088	0.097	0.000 (−0.003–0.003)	0.846	0.294	0.396
GCL	−0.001 (− 0.004–0.002)	0.421	0.274	0.559	−0.003 (− 0.006 – − 0.001)	**0.010**	0.931	0.931
IPL	-0.001 (−0.003–0.002)	0.649	0.156	0.468	− 0.002 (− 0.004–0.000)	0.074	0.447	0.502
INL	−0.001 (− 0.003–0.001)	0.407	0.719	0.863	−0.002 (− 0.004–0.000)	**0.025**	0.519	0.549
OPL	− 0.002 (− 0.004–0.000)	0.100	0.333	0.510	−0.003 (− 0.004 – − 0.002)	**< 0.001**	**0.002**	**0.005**
ONL	0.000 (− 0.006–0.007)	0.890	0.338	0.559	− 0.007 (− 0.011 – − 0.002)	**0.008**	**0.015**	**0.033**
Whole	− 0.001 (− 0.016–0.013)	0.859	0.245	0.364	−0.020 (− 0.031– − 0.009)	**< 0.001**	**0.040**	**0.047**
Subfoveal choroid	− 0.183 (− 0.267 – − 0.098)	**< 0.001**	**0.004**	**0.007**	−0.194 (− 0.257 – − 0.132)	**< 0.001**	**< 0.001**	**< 0.001**

*PPA* Parapapillary atrophy, *PPA*_*+BM*_ β-parapapillary atrophy with Bruch’s membrane, *PPA*_*-BM*_ β-parapapillary atrophy without Bruch’s membrane, *B* Unstandardized coefficient, *NFL* Nerve fiber layer, *GCL* Ganglion cell layer, *IPL* Inner plexiform layer, *INL* Inner nuclear layer, *OPL* Outer plexiform layer; *ONL* Outer nuclear layer

^a^*P* value adjusted for age and sex

^b^*P* value adjusted for age, sex, and axial length

^c^*P* value adjusted for age, sex, and axial length. They were adjusted with Benjamini-Hochberg procedure

Values with statistical significance are shown in bold

## Discussion

In this study, we evaluated the association of each macular layer thickness with axial length and the microstructure of PPA in myopic eyes. We found that central macular thickness positively correlated with axial length, whereas peripheral macular thickness and subfoveal choroidal thickness were negatively associated with axial length. Our study also revealed that PPA_+BM_ width had no association with any macular layer thickness. However, PPA_-BM_ width had correlations with many macular layer thicknesses. Moreover, both PPA_+BM_ and PPA_-BM_ widths were negatively correlated with subfoveal choroidal thickness. To our knowledge, this is the first study demonstrating the association between macular thickness and the microstructure of PPA in myopic eyes.

In terms of regional changes, our study showed that macular thickness increased in the central region, but was stable in the pericentral region and decreased in the peripheral region with a more obvious change in the longer eye. This finding is in accordance with those of previous studies [8, 34–36]. The thickening of the central region might be due to the elongation of the eyeball, which was, in turn, caused by mechanical traction of the sclera. In such a condition, the extension of the sclera drives retinal thinning. Meanwhile, the tendency of the ILM to remain flat and the centripetal force of the posterior vitreous lead to the thickening of the central region [37].

In our study, the thickening of the central region was mainly attributed to the increase in OPL thickness, which was formed by dendrites [30]. Our result was consistent with that of a recent SD-OCT study on retinal thickness [38]. We speculate that the OPL comprises thousands of dendrites, and is more elastic than the ONL. As the axial length increases, the elevation of the central macular region would result in OPL deformation. However, some studies have reported different results about the correlations between macular thickness and axial length. The different study design and inclusion criteria might explain these conflicting results. For instance, in the study of Wakitani et al. [39] OCT scans were centered on the fovea with a scan length of 3 mm, while the scan length was 6 mm in our study. High myopia was excluded from the study by Ooto et al. [27], and hyperopia was included in the study by Song et al. [40], while high myopia was included and hyperopia was excluded in our study. It is possible that the changes in retinal thickness in low-to-moderate myopia may be not enough to induce a morphological alteration.

In the peripheral region, the correlation between macular thickness and axial length was strong. All intraretinal layer thicknesses were correlated with axial length, and the thinning of the peripheral region was mainly because of the thinning of the GCL, IPL, and INL. This tendency was reported by Harb et al. [41], who stated that macular thinning in myopic eyes was more obvious in the peripheral region than in the other 2 regions.

In this study, RNFL thickness had a positive correlation with axial length in the pericentral and peripheral regions. Similar results had been described by Kim et al. [42] They proposed that with the expansion of the posterior pole, the retina could be pulled toward the temporal side, and in the RNFL, the compressed fiber bundles from the hemisphere on either side would be dragged to the horizontal raphe.

In terms of β-zone PPA, PPA_+BM_ has been reported to be associated with older age or myopia [3–5]. But previous investigations have also revealed that PPA_+BM_ was more significantly correlated with glaucoma [1, 2, 4, 43]. Teng et al. [44] described that high IOP in patients with glaucoma may cause obstruction of the parapapillary choriocapillaris and, in turn, may lead to the degeneration of the RPE and adjacent cells. Similarly, Sullivan-Mee et al. [45] reported a significant correlation between juxtapapillary choroidal volume and PPA_+BM_. The loss of RPE in the PPA_+BM_ could be caused by disturbance of blood supply due to a thinned choroid, suggesting that PPA_+BM_ may be associated with vascular compromise.

Conversely, PPA-_BM_ has been known to be mainly associated with axial elongation. Recent studies showed that an increase in axial length could lead to remodeling of the parapapillary region, and the backward pull via the optic nerve may act on the posterior sclera, leading to the formation and development of PPA_-BM_ [3, 46, 47]. Lee et al. [48] reported that the development of PPA_-BM_ reflected scleral overgrowth when compared with the inner retinal structures in the growing eye. Hence, PPA_-BM_ is strongly correlated with axial length, and this might account for the association between PPA_-BM_ and myopia. Chui et al. [49] proved that retinal extension may not mirror scleral growth when measuring 2 parameters of the posterior pole, and the retina could slide to the temporal side during eye growth. Collectively, we suggest that PPA_+BM_ is primarily involved in localized impairment of parapapillary choriocapillaris circulation and that PPA_-BM_ mainly reflects the broad posterior pole change caused by optic nerve traction due to axial elongation.

Multiple linear regression analysis revealed that PPA_+BM_ width had no association with macular layer thickness. However, the relationship between PPA_-BM_ width and macular layer thickness was obvious. In addition, some macular intraretinal layer thicknesses had a stronger correlation with PPA_-BM_ width than with axial length. This result led us to presume that as a monitoring marker, PPA_-BM_ might reflect changes of macular microstructure. Many studies had shown that the macular thickness reduced in glaucoma patient. Assessment of macular thickness can give us the valuable information for the diagnosis and management of glaucoma. However, our study implied that some intraretinal layers and whole macula can be thinner in myopia with large PPA_-BM_ width who was non-glaucoma patient. Therefore, PPA_-BM_ should be considered when evaluating macula in myopia and glaucoma patient.

The choroids can be thinner as the axial length increases [10, 11]. In our study, subfoveal choroidal thickness was significantly correlated with both PPA_+BM_ and PPA_-BM_ widths. Interestingly, PPA-_BM_ width was correlated with both subfoveal choroidal thickness and macular thickness. Nevertheless, PPA_+BM_ width was correlated with subfoveal choroidal thickness, but not with macular thickness. The development of PPA-_BM_ reflects that axial elongation and broad posterior pole changes may be involved in the sclera, choroid, and retina. Hence, PPA-_BM_ is associated with both subfoveal choroidal thickness and macular thickness. As mentioned above, PPA_+BM_ could be associated with disturbance of blood supply due to a thinned choroid, not with retina. That might explain why PPA_+BM_ was correlated with choroid, but not with macula.

Our study has several limitations. First, we could not account for optic disc tilt, which may be an important measurement indicator of the ONH. Previous studies have shown that optic disc tilt is correlated with the perfusion of the foveal zone [50] and RNFL thickness [51]. A more important association between optic disc tilt and macular thickness may exist. Second, observing the changes of the equatorial retina in detail was difficult, but knowledge about this could let us further understand the progression trend of myopia. Finally, we did not measure the lamina cribrosa and sclera. For understanding the underlying mechanism of myopia, future studies should assess the lamina cribrosa and sclera in myopic eyes.

## Conclusions

In conclusion, our study showed that macular intraretinal layer thicknesses have significant associations with axial length and PPA-_BM_ width. With an increase in axial length, whole macular thickness in the peripheral region decreased, while whole macular thickness in the central region increased. PPA-_BM_ width was correlated with several macular intraretinal layer thicknesses; however, no correlation was observed between PPA_+BM_ width and macular intraretinal layer thickness. Given the clinical significance of macular evaluation in managing glaucoma, the microstructure of PPA, especially PPA-_BM_, should be considered when evaluating the macula in patient with myopia and glaucoma.

## Data Availability

The datasets analyzed in this study are available from the corresponding author (Sang Woo Park, exo70@naver.com) upon reasonable request.

## Abbreviations

BCVA: Best corrected visual acuity
BM: Bruch’s membrane
BMO: Bruch’s membrane opening
EDI: Enhanced depth imaging
GCL: Ganglion cell layer
ILM: Internal limiting membrane
INL: Inner nuclear layer
IOP: Intraocular pressure
IPL: Inner plexiform layer
IR: Infrared
mGCIPL: Macular ganglion cell-inner plexiform layer
ONH: Optic nerve head
ONL: Outer nuclear layer
OPL: Outer plexiform layer
PPA: Parapapillary atrophy
PPA_+BM_: β-parapapillary atrophy with Bruch’s membrane
PPA-_BM_: β-parapapillary atrophy without Bruch’s membrane
pRNFL: Parapapillary retinal nerve fiber layer
RNFL: Retinal nerve fiber layer
RPE: Retinal pigment epithelium
SD-OCT: Spectral-domain optical coherence tomography
SE: Spherical equivalent
